# Compliment to Gen. Taylor by the New York State Society

**Published:** 1849-04

**Authors:** 


*Compliment to Gen. Taylor by the New York State Society.*—Doctor Stevens, President of the State Society, at the last annual meeting, proposed a recommendation, by the Society, of Gen. Taylor, to the Regents of theUniversity, for the honorary degree of M. D. The following resolutions, which were offered by Dr. F. H. Hamilton, were adopted as a substitute for the diploma, for which substitution we presume Gen. Taylor will be much obliged:—

*Resolved*, That the sentiment uttered by Gen. Zachary Taylor, at the battle of Buena Vista, that "He would not leave his sick and wounded behind him," is eminently humane, and merits a record and memorial in the archives of the medical profession.

*Resolved*, That it is hereby recorded and commemorated.

*Resolved*, That a copy of this resolution, signed by the President and Secretary, be transmitted to Gen. Taylor.

